# The Association of Sport Performance with *ACE* and *ACTN3* Genetic Polymorphisms: A Systematic Review and Meta-Analysis

**DOI:** 10.1371/journal.pone.0054685

**Published:** 2013-01-24

**Authors:** Fang Ma, Yu Yang, Xiangwei Li, Feng Zhou, Cong Gao, Mufei Li, Lei Gao

**Affiliations:** 1 The Kinesiology Laboratory, Physical Education Institute, Xinjiang Normal University, Urumqi, China; 2 MOH Key Laboratory of Systems Biology of Pathogens, Institute of Pathogen Biology, Chinese Academy of Medical Sciences & Peking Union Medical College, Beijing, China; University of Buenos Aires, Cardiovascular Pathophysiology Institute, Argentina

## Abstract

**Background:**

Genetic polymorphism is suggested to be associated with human physical performance. The angiotensin I-converting enzyme insertion/deletion (*ACE* I/D) polymorphism and the α-actinin-3 gene (*ACTN3*) R577X polymorphism have been most widely studied for such association analysis. However, the findings are frequently heterogeneous. We aim to summarize the associations of *ACE* I/D and *ACTN3* R577X with sport performance by means of meta-analysis.

**Methods:**

We systematically reviewed and quantitatively summarized published studies, until October 31, 2012, on relationship between *ACE/ACTN3* genetic polymorphisms and sports performance, respectively.

**Results:**

A total of 366 articles on *ACE* and 88 articles on *ACTN3* were achieved by literature search. A significant association was found for ACE II genotype compared to D allele carriage (DD+ID) with increased possibility of physical performance (OR, 1.23; 95% CI, 1.05–1.45). With respect to sport discipline, the II genotype was found to be associated with performance in endurance athletes (OR, 1.35; 95% CI, 1.17–1.55). On the other hand, no significant association was observed for *ACTN3* RR genotype as compared to X allele carriage (XX+RX) (OR, 1.03; 95% CI, 0.92–1.15). However, when restricted the analyses to power events, a significant association was observed (OR, 1.21; 95% CI, 1.03–1.42).

**Conclusion:**

Our results provide more solid evidence for the associations between *ACE* II genotype and endurance events and between *ACTN3* R allele and power events. The findings suggest that the genetic profiles might influence human physical performance.

## Introduction

Elite athletes are defined as the one who has competed at a national or international level in a given sport [1]. The concept that genetic traits are strongly associated with human physical performance has been wildly accepted in the past decade. For instance, it was suggested that the heritability of athlete status was estimated at approximately 66% [2]. Researchers are now concentrating on looking for the exact genetic profiles contribute to sport performance and determining the underlying mechanisms involved in specific fields of elite athletic performance. One of the main aims of such studies is to help clinicians and coaches to recognize and guide individuals with genetic potentiality to be elite athletes.

Here, we specifically consider two genes which have been extensively studied for the association with athletic ability, namely, the angiotensin I-converting enzyme (ACE) and α-actinin-3 (ACTN3). The first evidence of genetic polymorphisms influencing human physical performance is reported for *ACE* gene [3], [4]. The *ACE* insertion/deletion (ACE I/D, rs1799752) polymorphism has been related with improvements in performance and exercise duration in a variety of populations. The I allele, which represents an insertion of 287 bp, is associated with lower serum [5] and tissue [6] ACE activity and improved performance in endurance sports. The deleted form of the variant (D allele) is associated with higher circulating and tissue ACE activity [7] and enhanced performance at sports requiring sprinting or short bursts of power. *ACTN3* has also been well studied as a target gene. The *ACTN3* gene encodes the protein α -actinin-3, which is almost exclusively expressed to sarcomere of the fast glycolytic type II fibers that are responsible for the generation of rapid forceful contractions during activities such as sprinting and weightlifting [8], [9]. A genetic variation in the *ACTN3* gene that results in the replacement of an arginine (R) with a stop codon (X) at amino acid 577 (R577X, rs1815739) can create two different versions of the *ACTN3* gene. Both of these two versions are common in the general population. However, the findings on the relations between genetic polymorphisms and sports performance are frequently heterogeneous.

In this article, we aim to summarize the associations of sport performance with *ACE* and *ACTN3* genetic polymorphisms by means of meta-analysis, which might provide more solid evidence as compared with individual reports.

## Results

**Figure 1:**
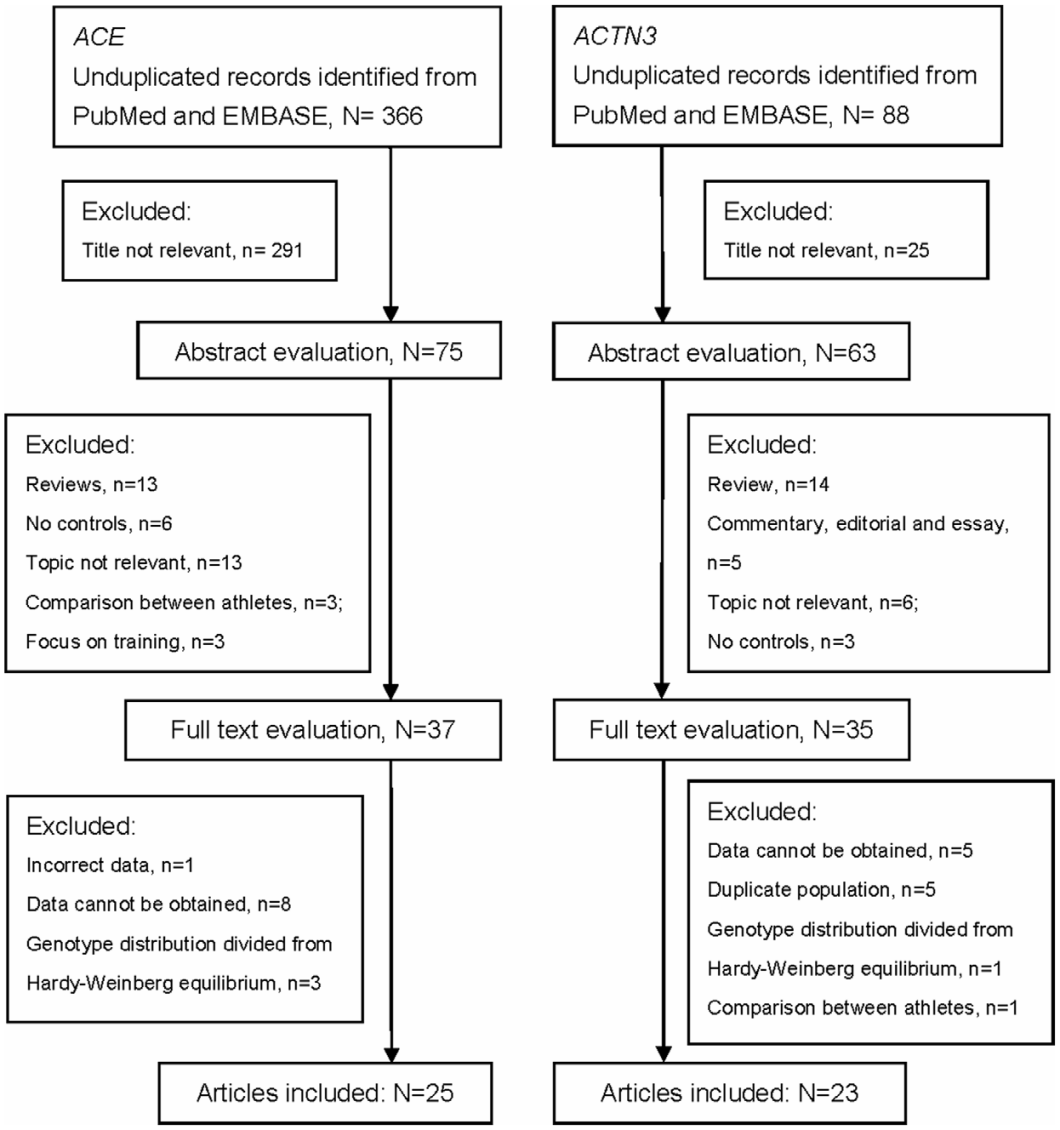
Flow Diagram of study design.

After excluding the overlapped results between the databases, a total of 366 articles about *ACE* and 88 articles focused on *ACTN3* were achieved by literature search separately, from PubMed and EMBASE, using different combination of key terms. As shown in **Figure 1**, the articles on *ACE* or *ACTN3* were screened separately. After excluding papers whose topics are not relevant, 75 abstracts and 63 abstracts were retrieved for next step. After abstract evaluation, 37 studies addressing the association of ACE polymorphisms and sport performance, and 35 studies addressing the association of *ACTN3* polymorphisms and sport performance were identified for detailed full text evaluation. Finally, 25 articles addressing *ACE* and 23 articles addressing *ACTN3* were included in this study, respectively. Among them, there are 6 articles reported data on both *ACE* and *ACTN3*. Please refer to **Table S1** for more detailed information on study identification.

As shown in **Table S2**, not all included articles provided necessary information for sub-group analysis. Among the 25 articles provided information of *ACE* I/D polymorphism, 10 studies were included in gender sub-group analysis, 17 studies and 13 studies were included in endurance and power groups for sport discipline sub-group analysis, respectively. In the 23 articles provided data of *ACTN3* R577X polymorphism, 13 studies were included in gender sub-group analysis, 15 studies and 18 studies were included in endurance and power groups for sport discipline sub-group analysis, respectively.

**Figure 2:**
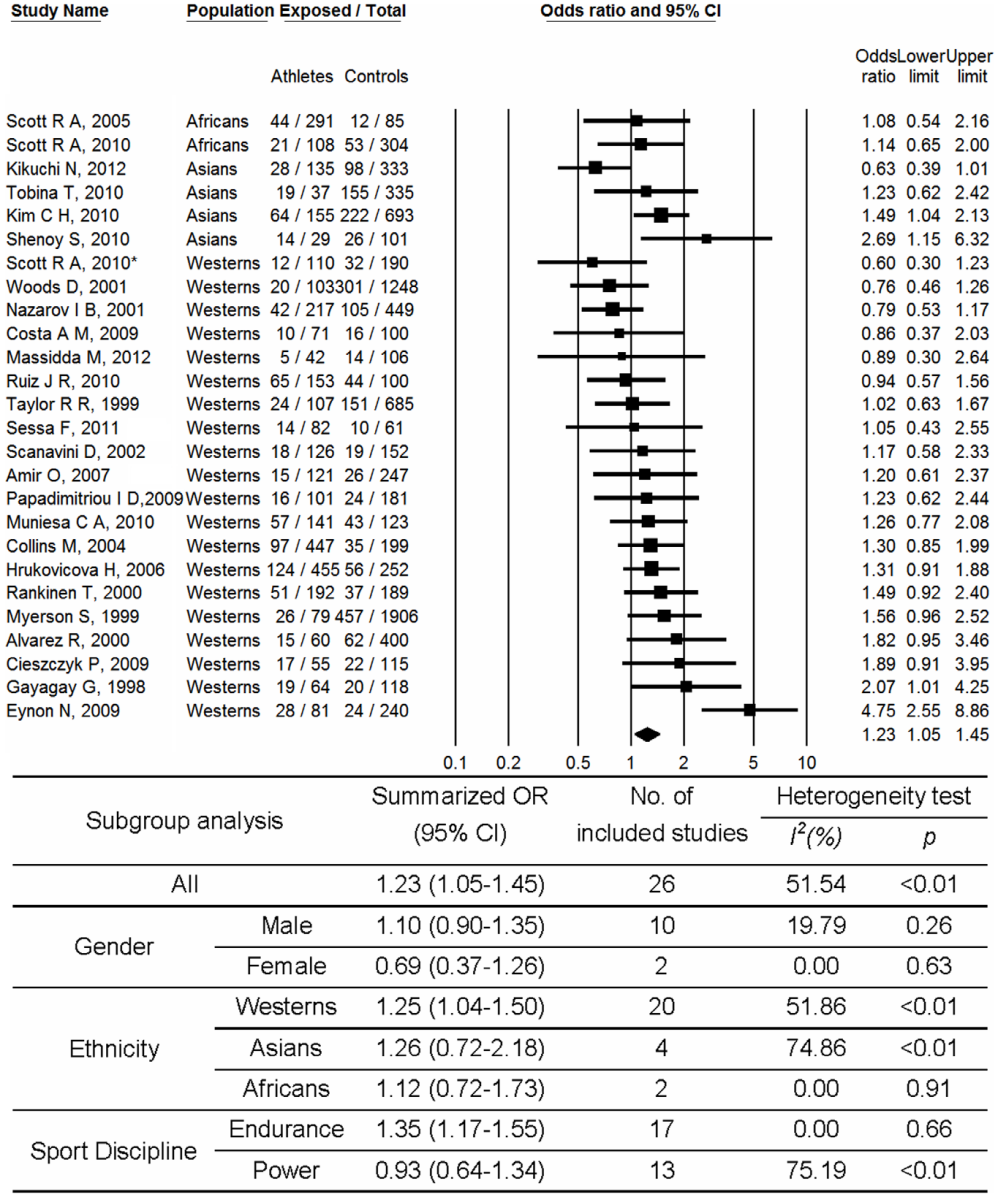
Meta-analysis of the association between sport performance and *ACE* polymorphism (II vs. ID+DD). Abbreviation: CI, confidence interval; OR, odds ratio. *Different study population from the same article.

**Figure 3:**
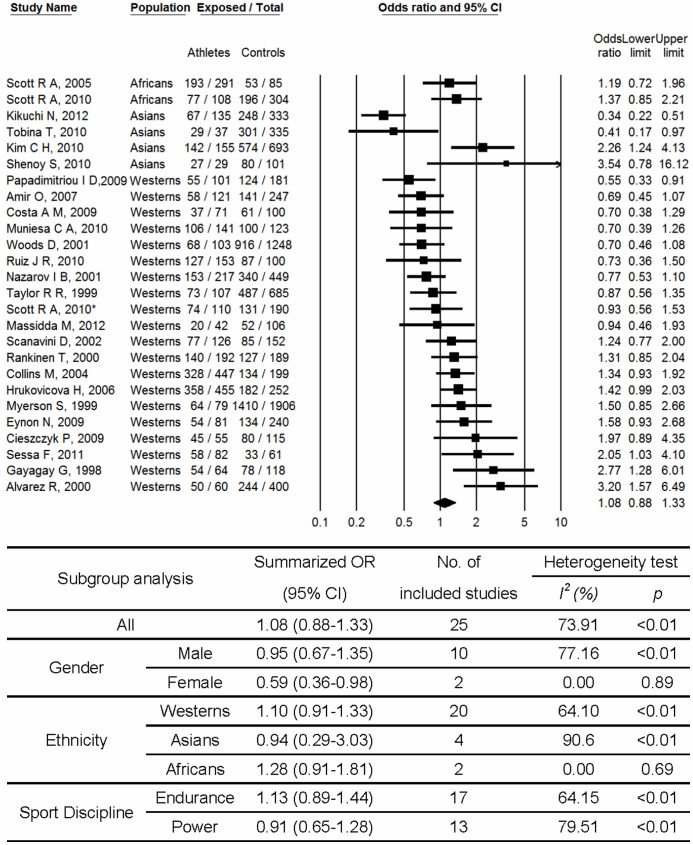
Meta-analysis of the association between sport performance and *ACE* polymorphism (II+ID vs. DD). Abbreviation: CI, confidence interval; OR, odds ratio. *Different study population from the same article.

As shown in **Figure 2**, a significant association was found for *ACE* II genotype compared to D allele carriage (DD+ID) with increased possibility of physical performance (OR, 1.23; 95% CI, 1.05–1.45). Medium heterogeneity between studies (p<0.01; I^2^ = 51.54%) was observed. No significant publication bias was observed (p = 0.96 for Begg rank correlation analysis; p = 0.59 for Egger weighted regression analysis). In the subgroup analysis with respect to gender, no significant relationship was observed for males (OR, 1.10; 95% CI, 0.90–1.35) and females (OR, 0.69; 95%CI, 0.37–1.26). In the subgroup analysis with respect to ethnicity, significant relationships were observed for Westerns (OR, 1.25; 95%CI, 1.04–1.50). But with respect to sport discipline, the II genotype was found to be associated with performance in endurance athletes (OR, 1.35; 95% CI, 1.17–1.55) but not in power athletes (OR, 0.93; 95% CI, 0.64–1.34). When the analyses were based on a dominant model (**Figure 3**), I allele carrier (II+ID) was found to be associated with decreased sports performance in females (OR, 0.59; 95% CI, 0.36–0.98). And no significant publication bias was observed (p = 0.17 for Begg rank correlation analysis; p = 0.16 for Egger weighted regression analysis).

**Figure 4:**
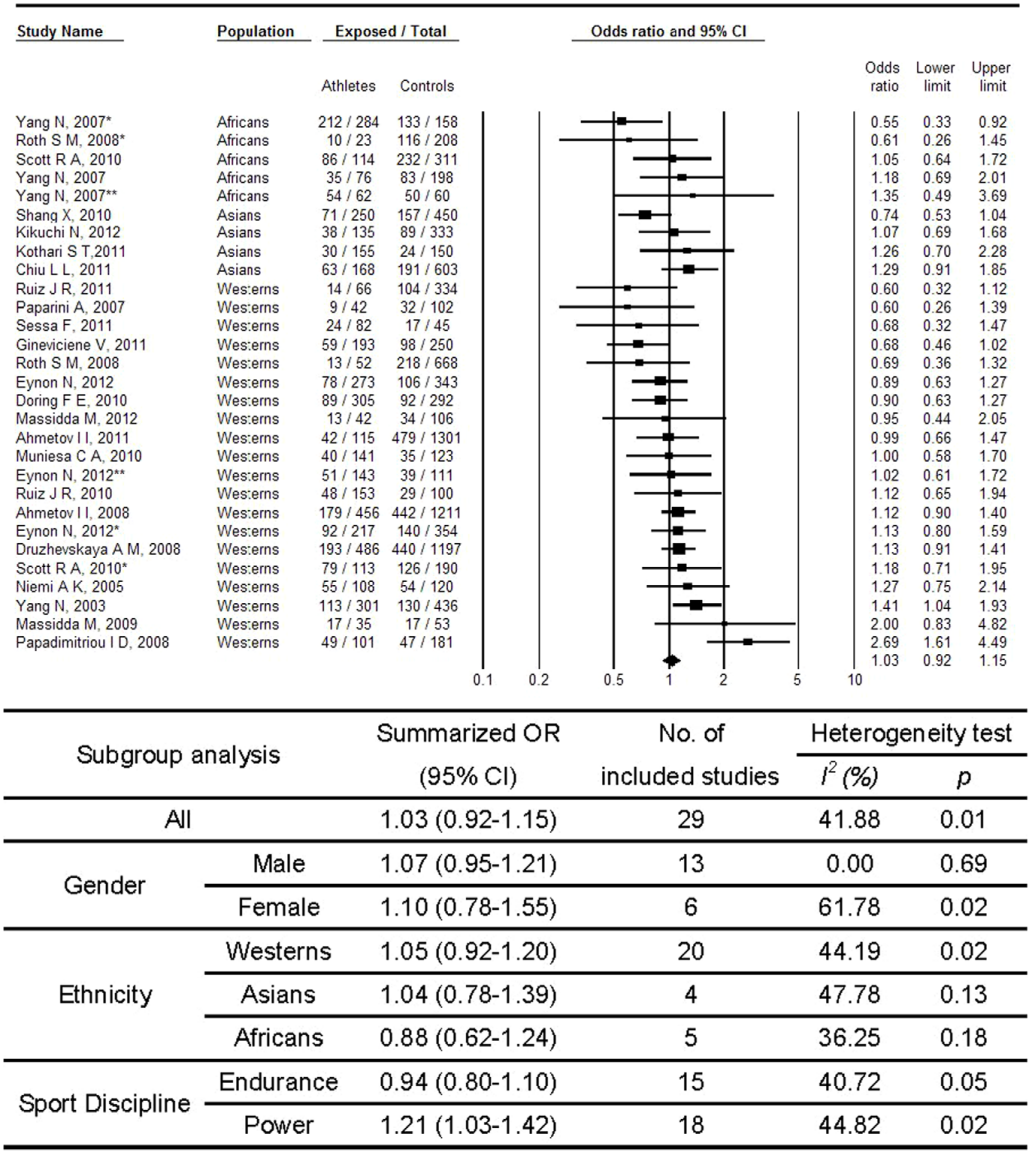
Meta-analysis of the association between sport performance and *ACTN3* polymorphism (RR vs. RX+XX). Abbreviation: CI, confidence interval; OR, odds ratio. *, **Different study population from the same article.

**Figure 5:**
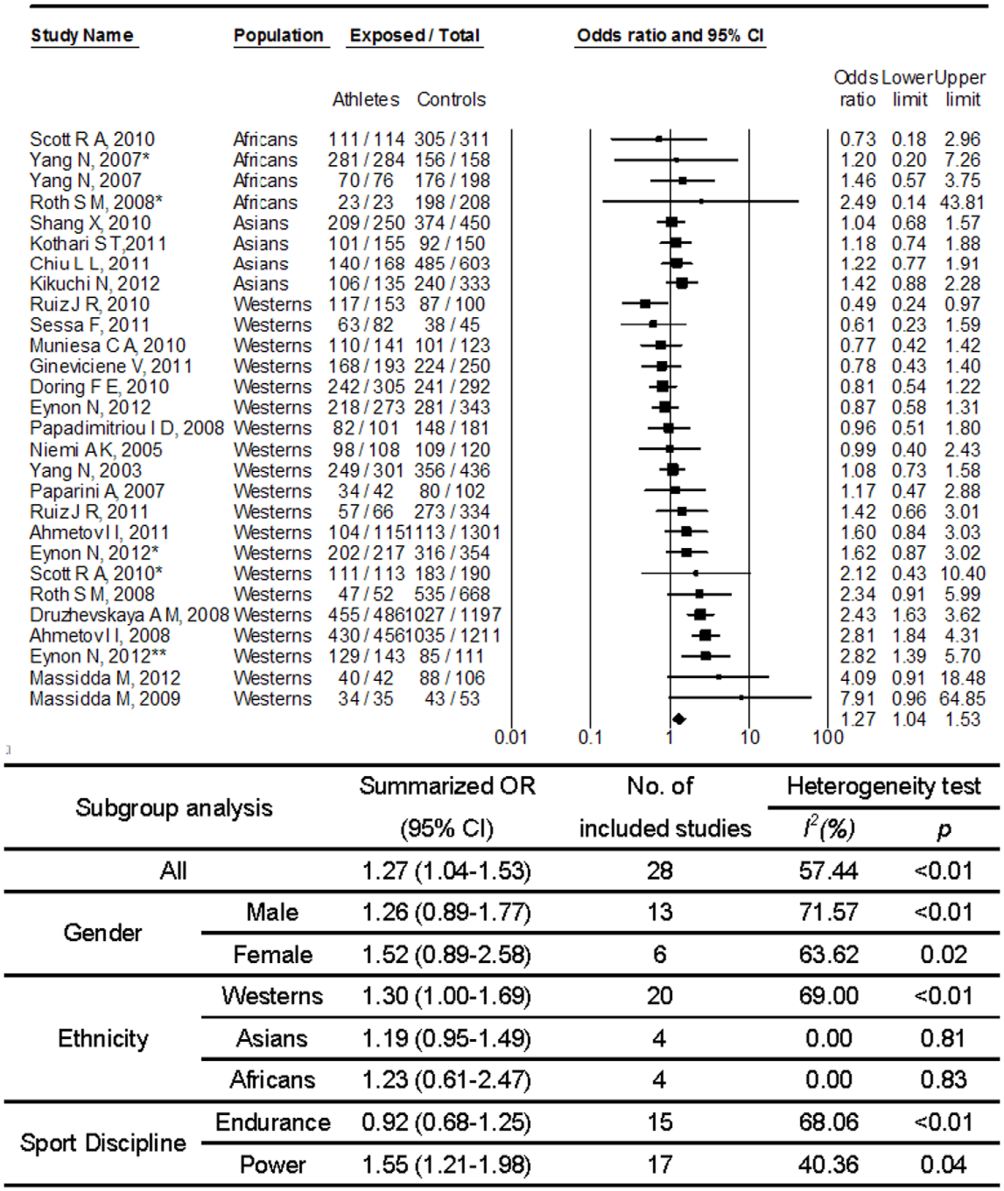
Meta-analysis of the association between sport performance and *ACTN3* polymorphism (RR+RX vs. XX). Abbreviation: CI, confidence interval; OR, odds ratio. *Different study population from the same article.

**Figure 4** shows the associations between *ACTN3* R577X and sport performance in a recessive model. No significant association was found for *ACTN3* RR genotype as compared to X allele carriage (XX+RX) (OR, 1.03; 95% CI, 0.92–1.15). Heterogeneity between studies was medium (p = 0.01; I^2^ = 41.88%). No substantial publication bias was observed (p = 0.42 for Begg rank correlation analysis; p = 0.38 for Egger weighted regression analysis). When restricted the analyses to power events, a significant association was observed (OR, 1.21; 95% CI, 1.03–1.42). In the analyses based on dominant model as shown in **Figure 5**, R allele carrier was consistently associated with increase possibility of sports performance among power events (OR, 1.55; 95% CI, 1.21–1.98). And again, no substantial publication bias was observed (p = 0.16 for Begg rank correlation analysis; p = 0.59 for Egger weighted regression analysis).

## Discussion

This review estimated the association of human sport performance with *ACE* I/D and ACTN3 R577X by means of meta-analysis. Significant relations were observed between *ACE* II genotype and endurance events, and *ACTN* R allele and power events, respectively. Subgroup analyses suggest gender, ethnicity and sport discipline might explain, at least in part, the existing heterogeneity between included studies.

It has been accepted that a number of elite athletes were natural. Athletes might be inherently predisposed towards specialist performance in one area. A vast array of human phenotypes was suggested to influence sports performance, such as muscle strength, skeletal structure, tendon elasticity, and heart and lung size. These phenotypes themselves are influenced by a variety of other processes and cellular pathways which are eventually influenced by a large number of individual and relevant genes. The *ACE* I/D polymorphism in intron 16 affects the function of the gene, differentiating the enzymatic activity of angiotensin convertase in the blood [10], [11], which is connected with the regulation of blood pressure and as such, it plays an important role in cardiorespiratory efficiency [12], [13]. The distributions of the three variants (II, ID, DD) within a Caucasian population are roughly 25%, 50%, and 25% respectively [14]. And those are not remarkable different from Asian population in Korea (23%, 66% and 11% respectively) [15]. Followed by Montgomery et al. who demonstrated the relationship between the *ACE* polymorphism and sport performance [3], Gayagay et al. first found a significant excess of the I allele and the II genotype in Australian national rowers attending their pre-Olympics selection trial [4]. Generally, the I allele seems associated with endurance-orientated events, while the D allele seems like to be the opposite with power-orientated events [16]–[21]. Plenty of studies were performed to support the theory. Cieszczyk et al. reported that a significantly different I allele frequency between rowers and controls in Poland population, which indicated positive association of the I allele with endurance performance [20]. In a study conducted among 495 respondents who were potential Olympic competitors identified by the British Olympic Association, 91 runners were found carrying a significant excess of both I allele (p = 0.01) and II genotype (p = 0.019) as compared with controls [22]. Examination of the gene frequency within a single sporting discipline with a spectrum from power-orientated short, to more endurance-based longer distances is a preferred strategy. Variety of studies have employed this strategy and consistently found the association between *ACE* I allele and longer distance sport events [23], [24]. However, there are also some exceptions. Amir, O and colleagues reported that the frequency of the *ACE* D allele and *ACE* DD genotype in Israeli elite marathon runners seems to be higher than in sprinters, which suggested a positive association between the D allele and the likelihood of being an elite endurance athlete in some ethnic groups [25]. In our present meta-analysis, there was no statistically significant association between *ACE* I allele and endurance sport events, but it was very close to be so (OR, 1.13; 95% CI, 0.89–1.44). Moreover, a significant association between *ACE* II genotype and endurance group was observed (OR, 1.35; 95% CI, 1.17–1.55). These results may suggest for larger population and more specific studies in different ethnic groups.

The *ACTN3* gene encodes for the synthesis of a-actinin-3 in skeletal-muscle fibres, a sarcomeric protein necessary for producing ‘explosive’ powerful contractions. A premature stop codon polymorphism in *ACTN3* was first described by North and colleagues [26]. In 2003, Yang et al. demonstrated a significant association between *ACTN3* genotype and athletic performance [27]. They found that both male and female elite sprint athletes have significantly higher frequencies of the 577R allele compare to controls. Thus, unlike *ACE* I/D, researchers generally concentrate on association between *ACTN3* R577X and power events. Some articles have consistently reported a strong association between RR genotype and elite power performance [28]–[31]. The results of stratified analyses of power events in our present study are consistent with these studies as well (OR, 1.55; 95% CI, 1.21–1.98). As *ACTN3* R allele was suggested to be associated with power performance, *ACTN3* XX might be postulated to contribute to endurance performance theoretically. However, reports from Asians and Africans suggested that *ACTN3* deficiency might not associated with endurance performance [32], [33].

With respect to the rapid increase in the number of original researches in this area, review articles have been published from different perspectives [34]–[36]. Montgomery group published a review on *ACE* I/D polymorphism research published during 1998–2010 which suggests that the I allele is tending to be associated with endurance sports [34]. More recently, Zilberman-Schapira conducted a literature survey on sports and genes and discussed the important issues on methodology of such studies [36]. However, these reviews did not verify their findings by quantitative analysis in this review. Tamuno and colleagues conducted a meta-analysis on the published association between *ACTN3* and athletic status up to November 29, 2010 and observed an overrepresentation of the *ACTN3* R577X RR genotype in power athletes in Europeans [35]. However, original article reported departure of Hardy-Weinberg Equilibrium (HWE) in the control group was not excluded from this meta-analysis, which might introduce selection bias into the summarized results. In addition, there are 12 articles on this topic were newly published in the past two years and were included in our update meta-analysis. Although the major findings were not substantially changed, our results provided more solid evidence for the relationship between *ACTN3* R577X genotype and sport performance.

There are some limitations of this systematic review that should be kept in mind. First, the potential confounding effect of performance level was not considered in present study because of the different criterions of elite athletes. Second, because not all necessary information could be obtained from all included studies, more detailed sub-analysis was limited. For example, the crude division of ethnics groups into ‘Asian’, ‘African’ and ‘Western’ may make the analyses be prone to bias. Therefore, further studies from different populations are warranted to verify the current findings. Third, only articles provided data on both athletes and sedentary controls were included in this present study, which might introduce potential selection bias. Fourth, although studies reporting negative genetic association are less likely to be published than others showing ‘statistical significance’ (‘positive results’), no evident publication bias was observed in our present analyses (p>0.05). Finally, due to the variety of definition of endurance/power events and some studies reported data of mixed sport disciplines, phenotypic heterogeneity cannot be excluded.

In conclusion, the present study summarized the associations of sport performance with *ACE* I/D and *ACTN3* R577X polymorphisms. The results consistently provided more solid evidence for associations between *ACE* II genotype and endurance events, and between *ACTN* R allele and power events. Our findings provided more solid evident to support that human physical performance might be influenced by genetic profiles.

## Materials and Methods

### Literature Identification

Studies addressing relations between *ACE*/*ACTN3* genetic polymorphisms and sports performance were identified by searching for published original articles in PubMed (1946- ) and EMBASE (1974- ) until October 31, 2012. Combinations of the key words “sport” and “ACTN3” or “alpha-actinin-3” were used to screen for potentially relevant studies focused on *ACTN3*. Combinations of the key words “sport” and “ACE” or “angiotensin-converting enzyme” were used to screen for potentially relevant studies focused on *ACE*. Additional studies were also identified by cross-referencing [37].

### Inclusion and Exclusion Criteria

Original articles presented case-control or cohort studies on human and published in English were considered. Articles reported the distribution of single nucleotide polymorphism of *ACE* or *ACTN3* among both athletes and sedentary health controls were considered. If the study was reported in duplicate, the version firstly published was included. Short reports or letters were included if the distribution data of *ACE* or *ACTN3* could be obtained. Exclusion criteria were: (i) review articles, congress abstracts, commentaries or other unoriginal studies; (ii) studies reported in languages other than English; (iii) articles did not provide necessary data; (iv) departure from HWE was detected in a control group.

### Data Extraction

For all studies, we extracted the following data from original publications: first author and year of publication; distribution of genotypes for each polymorphism among athletes and controls, characteristics of the study design and the study population (gender and numbers of athletes and controls, sport disciplines and host ethnicity) We defined different sport disciplines into two divide category, as endurance and power and please refer to **Table S3** for the sport classify.

### Statistical Analysis

HWE was examined in controls by asymptotic Pearson’s chi-square test for each polymorphism in each study. The association between polymorphism and sport performance was estimated by means of odds ratios (OR) and corresponding 95% confidence intervals (CI) comparing athletes to controls. Meta-analyses were carried out using Comprehensive Meta-Analysis (V2.0, Biostat, Englewood, NJ, USA). Random effects models were used for meta-analysis, taking into account the possibility of heterogeneity between studies which was tested by the Q test and I^2^ test. Stratified analyses were conducted with respect to gender, sport discipline and host ethnicity. The latter was categorized as Africans, Asians and Westerns (Europeans and Americans). Because of the limited number of publications in Americans, they were sub-grouped to Westerns combined with studies from Europeans. Begg rank correlation method [38] and Egger weighted regression method [39] were used to statistically assess publication bias (p<0.05 was considered indicative of statistically significant publication bias) [40].

## Supporting Information

Table S1 Study identification: Included and excluded articles after full-text evaluation
(DOC)

Table S2 ACE & ACTN3 basic information: Basic information of included articles
(XLS)

Table S3 Sport discipline: Definition of sport discipline in included articles
(DOC)

## References

[1] Macarthur DG, North KN (2005) Genes and human elite athletic performance. Hum Genet 116: 331–339. 15726413 10.1007/s00439-005-1261-8

[2] De Moor MH, Spector TD, Cherkas LF, Falchi M, Hottenga JJ, et al (2007) Genome-wide linkage scan for athlete status in 700 British female DZ twin pairs. Twin Res Hum Genet 10: 812–820. 18179392 10.1375/twin.10.6.812

[3] Montgomery HE, Marshall R, Hemingway H, Myerson S, Clarkson P, et al (1998) Human gene for physical performance. Nature 393: 221–222. 9607758 10.1038/30374

[4] Gayagay G, Yu B, Hambly B, Boston T, Hahn A, et al (1998) Elite endurance athletes and the ACE I allele–the role of genes in athletic performance. Hum Genet 103: 48–50. 9737775 10.1007/s004390050781

[5] Rigat B, Hubert C, Alhenc-Gelas F, Cambien F, Corvol P, et al (1990) An insertion/deletion polymorphism in the angiotensin I-converting enzyme gene accounting for half the variance of serum enzyme levels. J Clin Invest 86: 1343–1346. 1976655 10.1172/JCI114844 PMC296868

[6] Danser AH, Schalekamp MADH, Bax WA, van den Brink AM, Saxena PR, et al (1995) Angiotensin-converting enzyme in the human heart: effect of the deletion/insertion polymorphism. Circulation 92: 1387–1388. 7664416 10.1161/01.cir.92.6.1387

[7] Thompson WR, Binder-Macleod SA (2006) Association of genetic factors with selected measures of physical performance. Phys Ther 86: 585–591. 16579674 PMC4090215

[8] Mills M, Yang N, Weinberger R, Vander WD, Beggs AH, et al (2001) Differential expression of the actin-binding proteins, alpha-actinin-2 and -3, in different species: implications for the evolution of functional redundancy. Hum Mol Genet 10: 1335–1346. 11440986 10.1093/hmg/10.13.1335

[9] MacArthur DG, North KN (2007) ACTN3: A genetic influence on muscle function and athletic performance. Exerc Sport Sci Rev 35: 30–34. 17211191 10.1097/JES.0b013e31802d8874

[10] Rigat B, Hubert C, Alhenc-Gelas F, Cambien F, Corvol P, et al (1990) An insertion/deletion polymorphism in the angiotensin I-converting enzyme gene accounting for half the variance of serum enzyme levels. J Clin Invest 86: 1343–1346. 1976655 10.1172/JCI114844 PMC296868

[11] Williams AG, Rayson MP, Jubb M, World M, Woods DR, et al (2000) The ACE gene and muscle performance. Nature 403: 614. 10.1038/35001141 10688186

[12] Cambien F, Poirier O, Lecerf L, Evans A, Cambou JP, et al (1992) Deletion polymorphism in the gene for angiotensin-converting enzyme is a potent risk factor for myocardial infarction. Nature 359: 641–644. 1328889 10.1038/359641a0

[13] Tiret L, Kee F, Poirier O, Nicaud V, Lecerf L, et al (1993) Deletion polymorphism in angiotensin-converting enzyme gene associated with parental history of myocardial infarction. Lancet 341: 991–992. 8096947 10.1016/0140-6736(93)91075-w

[14] Jones A, Montgomery HE, Woods DR (2002) Human performance: a role for the ACE genotype? Exerc Sport Sci Rev 30: 184–190. 12398116 10.1097/00003677-200210000-00008

[15] Oh SD (2007) The distribution of I/D polymorphism in the ACE gene among Korean male elite athletes. J Sports Med Phys Fitness 47: 250–254. 17557068

[16] Nazarov IB, Woods DR, Montgomery HE, Shneider OV, Kazakov VI, et al (2001) The angiotensin converting enzyme I/D polymorphism in Russian athletes. Eur J Hum Genet 9: 797–801. 11781693 10.1038/sj.ejhg.5200711

[17] Tobina T, Michishita R, Yamasawa F, Zhang B, Sasaki H, et al (2010) Association between the angiotensin I-converting enzyme gene insertion/deletion polymorphism and endurance running speed in Japanese runners. J Physiol Sci 60: 325–330. 20574690 10.1007/s12576-010-0100-4 PMC10717577

[18] Shenoy S, Tandon S, Sandhu J, Bhanwer AS (2010) Association of Angiotensin Converting Enzyme gene Polymorphism and Indian Army Triathletes Performance. Asian J Sports Med 1: 143–150. 22375202 10.5812/asjsm.34855 PMC3289177

[19] Costa AM, Silva AJ, Garrido ND, Louro H, de Oliveira RJ, et al (2009) Association between ACE D allele and elite short distance swimming. Eur J Appl Physiol 106: 785–790. 19458960 10.1007/s00421-009-1080-z

[20] Cieszczyk P, Krupecki K, Maciejewska A, Sawczuk M (2009) The angiotensin converting enzyme gene I/D polymorphism in Polish rowers. Int J Sports Med 30: 624–627. 19455482 10.1055/s-0029-1202825

[21] Papadimitriou ID, Papadopoulos C, Kouvatsi A, Triantaphyllidis C (2009) The ACE I/D polymorphism in elite Greek track and field athletes. J Sports Med Phys Fitness 49: 459–463. 20087307

[22] Myerson S, Hemingway H, Budget R, Martin J, Humphries S, et al (1999) Human angiotensin I-converting enzyme gene and endurance performance. J Appl Physiol 87: 1313–1316. 10517757 10.1152/jappl.1999.87.4.1313

[23] Min SK, Takahashi K, Ishigami H, Hiranuma K, Mizuno M, et al (2009) Is there a gender difference between ACE gene and race distance? Appl Physiol Nutr Metab 34: 926–932. 19935855 10.1139/H09-097

[24] Tsianos G, Sanders J, Dhamrait S, Humphries S, Grant S, et al (2004) The ACE gene insertion/deletion polymorphism and elite endurance swimming. Eur J Appl Physiol 92: 360–362. 15138837 10.1007/s00421-004-1120-7

[25] Amir O, Amir R, Yamin C, Attias E, Eynon N, et al (2007) The ACE deletion allele is associated with Israeli elite endurance athletes. Exp Physiol 92: 881–886. 17631516 10.1113/expphysiol.2007.038711

[26] North KN, Yang N, Wattanasirichaigoon D, Mills M, Easteal S, et al (1999) A common nonsense mutation results in alpha-actinin-3 deficiency in the general population. Nat Genet 21: 353–354. 10192379 10.1038/7675

[27] Yang N, MacArthur DG, Gulbin JP, Hahn AG, Beggs AH, et al (2003) ACTN3 genotype is associated with human elite athletic performance. Am J Hum Genet 73: 627–631. 12879365 10.1086/377590 PMC1180686

[28] Chiu LL, Wu YF, Tang MT, Yu HC, Hsieh LL, et al (2011) ACTN3 genotype and swimming performance in Taiwan. Int J Sports Med 32: 476–480. 21472630 10.1055/s-0030-1263115

[29] Eynon N, Duarte JA, Oliveira J, Sagiv M, Yamin C, et al (2009) ACTN3 R577X polymorphism and Israeli top-level athletes. Int J Sports Med 30: 695–698. 19544227 10.1055/s-0029-1220731

[30] Papadimitriou ID, Papadopoulos C, Kouvatsi A, Triantaphyllidis C (2008) The ACTN3 gene in elite Greek track and field athletes. Int J Sports Med 29: 352–355. 17879893 10.1055/s-2007-965339

[31] Paparini A, Ripani M, Giordano GD, Santoni D, Pigozzi F, et al (2007) ACTN3 genotyping by real-time PCR in the Italian population and athletes. Med Sci Sports Exerc 39: 810–815. 17468578 10.1097/mss.0b013e3180317491

[32] Shang X, Huang C, Chang Q, Zhang L, Huang T (2010) Association between the ACTN3 R577X polymorphism and female endurance athletes in China. Int J Sports Med 31: 913–916. 20936592 10.1055/s-0030-1265176

[33] Yang N, MacArthur DG, Wolde B, Onywera VO, Boit MK, et al (2007) The ACTN3 R577X polymorphism in East and West African athletes. Med Sci Sports Exerc 39: 1985–1988. 17986906 10.1249/mss.0b013e31814844c9

[34] Puthucheary Z, Skipworth JR, Rawal J, Loosemore M, Van Someren K, et al (2011) The ACE gene and human performance: 12 years on. Sports Med 41: 433–448. 21615186 10.2165/11588720-000000000-00000

[35] Alfred T, Ben-Shlomo Y, Cooper R, Hardy R, Cooper C, et al.. (2011) ACTN3 genotype, athletic status, and life course physical capability: meta-analysis of the published literature and findings from nine studies. Hum Mutat. 10.1002/humu.21526 PMC3174315 21542061

[36] Zilberman-Schapira G, Chen J, Gerstein MB (2012) On Sports And Genes. Recent Pat DNA Gene Seq. 10.2174/187221512802717367 22762737

[37] Yang Y, Li X, Zhou F, Jin Q, Gao L (2011) Prevalence of drug-resistant tuberculosis in mainland China: systematic review and meta-analysis. PLoS One 6: e20343. 21674033 10.1371/journal.pone.0020343 PMC3108589

[38] Begg CB, Mazumdar M (1994) Operating characteristics of a rank correlation test for publication bias. Biometrics 50: 1088–1101. 7786990

[39] Egger M, Davey SG, Schneider M, Minder C (1997) Bias in meta-analysis detected by a simple, graphical test. BMJ 315: 629–634. 9310563 10.1136/bmj.315.7109.629 PMC2127453

[40] Li X, Yang Y, Zhou F, Zhang Y, Lu H, et al (2011) SLC11A1 (NRAMP1) polymorphisms and tuberculosis susceptibility: updated systematic review and meta-analysis. PLoS One 6: e15831. 21283567 10.1371/journal.pone.0015831 PMC3026788

