# Supplementary material for: The Association of Sport Performance with ACE and ACTN3 Genetic Polymorphisms: A Systematic Review and Meta-Analysis
Source: PLoS One. 2013 Jan 24;8(1):e54685. doi: 10.1371/journal.pone.0054685 (PMC3554644; doi:10.1371/journal.pone.0054685)
Supplement: Table S1 — Study identification: Included and excluded articles after full-text evaluation (DOC) [file pone.0054685.s001.doc]

**Table S1. Included and excluded articles after full-text evaluation**

**Part 1, included article:**

*ACE*

1. Gayagay G, Yu B, Hambly B, Boston T, Hahn A, et al. (1998) Elite endurance athletes and the ACE I allele--the role of genes in athletic performance. Hum Genet 103: 48-50.
2. Myerson S, Hemingway H, Budget R, Martin J, Humphries S, et al. (1999) Human angiotensin I-converting enzyme gene and endurance performance. J Appl Physiol 87: 1313-1316.
3. Taylor RR, Mamotte CD, Fallon K, van Bockxmeer FM (1999) Elite athletes and the gene for angiotensin-converting enzyme. J Appl Physiol 87: 1035-1037.
4. Alvarez R, Terrados N, Ortolano R, Iglesias-Cubero G, Reguero JR, et al. (2000) Genetic variation in the renin-angiotensin system and athletic performance. Eur J Appl Physiol 82: 117-120.
5. Rankinen T, Wolfarth B, Simoneau JA, Maier-Lenz D, Rauramaa R, et al. (2000) No association between the angiotensin-converting enzyme ID polymorphism and elite endurance athlete status. J Appl Physiol 88: 1571-1575.
6. Nazarov IB, Woods DR, Montgomery HE, Shneider OV, Kazakov VI, et al. (2001) The angiotensin converting enzyme I/D polymorphism in Russian athletes. Eur J Hum Genet 9: 797-801.
7. Woods D, Hickman M, Jamshidi Y, Brull D, Vassiliou V, et al. (2001) Elite swimmers and the D allele of the ACE I/D polymorphism. Hum Genet 108: 230-232.
8. Scanavini D, Bernardi F, Castoldi E, Conconi F, Mazzoni G (2002) Increased frequency of the homozygous II ACE genotype in Italian Olympic endurance athletes. Eur J Hum Genet 10: 576-577.
9. Collins M, Xenophontos SL, Cariolou MA, Mokone GG, Hudson DE, et al. (2004) The ACE gene and endurance performance during the South African Ironman Triathlons. Med Sci Sports Exerc 36: 1314-1320.
10. Scott RA, Moran C, Wilson RH, Onywera V, Boit MK, et al. (2005) No association between Angiotensin Converting Enzyme (ACE) gene variation and endurance athlete status in Kenyans. Comp Biochem Physiol A Mol Integr Physiol 141: 169-175.
11. Hruskovicova H, Dzurenkova D, Selingerova M, Bohus B, Timkanicova B, et al. (2006) The angiotensin converting enzyme I/D polymorphism in long distance runners. J Sports Med Phys Fitness 46: 509-513.
12. Amir O, Amir R, Yamin C, Attias E, Eynon N, et al. (2007) The ACE deletion allele is associated with Israeli elite endurance athletes. Exp Physiol 92: 881-886.
13. Cieszczyk P, Krupecki K, Maciejewska A, Sawczuk M (2009) The angiotensin converting enzyme gene I/D polymorphism in Polish rowers. Int J Sports Med 30: 624-627.
14. Costa AM, Silva AJ, Garrido ND, Louro H, de Oliveira RJ, et al. (2009) Association between ACE D allele and elite short distance swimming. Eur J Appl Physiol 106: 785-790.
15. Eynon N, Alves AJ, Yamin C, Sagiv M, Duarte JA, et al. (2009) Is there an ACE ID - ACTN3 R577X polymorphisms interaction that influences sprint performance? Int J Sports Med 30: 888-891.
16. Papadimitriou ID, Papadopoulos C, Kouvatsi A, Triantaphyllidis C (2009) The ACE I/D polymorphism in elite Greek track and field athletes. J Sports Med Phys Fitness 49: 459-463.
17. Kim CH, Cho JY, Jeon JY, Koh YG, Kim YM, et al. (2010) ACE DD genotype is unfavorable to Korean short-term muscle power athletes. Int J Sports Med 31: 65-71.
18. Muniesa CA, Gonzalez-Freire M, Santiago C, Lao JI, Buxens A, et al. (2010) World-class performance in lightweight rowing: is it genetically influenced? A comparison with cyclists, runners and non-athletes. Br J Sports Med 44: 898-901.
19. Ruiz JR, Arteta D, Buxens A, Artieda M, Gomez-Gallego F, et al. (2010) Can we identify a power-oriented polygenic profile? J Appl Physiol 108: 561-566.
20. Scott RA, Irving R, Irwin L, Morrison E, Charlton V, et al. (2010) ACTN3 and ACE genotypes in elite Jamaican and US sprinters. Med Sci Sports Exerc 42: 107-112.
21. Shenoy S, Tandon S, Sandhu J, Bhanwer AS (2010) Association of Angiotensin Converting Enzyme gene Polymorphism and Indian Army Triathletes Performance. Asian J Sports Med 1: 143-150.
22. Tobina T, Michishita R, Yamasawa F, Zhang B, Sasaki H, et al. (2010) Association between the angiotensin I-converting enzyme gene insertion/deletion polymorphism and endurance running speed in Japanese runners. J Physiol Sci 60: 325-330.
23. Sessa F, Chetta M, Petito A, Franzetti M, Bafunno V, et al. (2011) Gene polymorphisms and sport attitude in Italian athletes. Genet Test Mol Biomarkers 15: 285-290.
24. Kikuchi N, Min SK, Ueda D, Igawa S, Nakazato K (2012) Higher Frequency of the Actn3 R Allele + Ace Dd Genotype in Japanese Elite Wrestlers. J Strength Cond Res.
25. Massidda M, Corrias L, Ibba G, Scorcu M, Vona G, et al. (2012) Genetic markers and explosive leg-muscle strength in elite Italian soccer players. J Sports Med Phys Fitness 52: 328-334.

*ACTN3*

1. Yang N, MacArthur DG, Gulbin JP, Hahn AG, Beggs AH, et al. (2003) ACTN3 genotype is associated with human elite athletic performance. Am J Hum Genet 73: 627-631.
2. Niemi AK, Majamaa K (2005) Mitochondrial DNA and ACTN3 genotypes in Finnish elite endurance and sprint athletes. Eur J Hum Genet 13: 965-969.
3. Paparini A, Ripani M, Giordano GD, Santoni D, Pigozzi F, et al. (2007) ACTN3 genotyping by real-time PCR in the Italian population and athletes. Med Sci Sports Exerc 39: 810-815.
4. Yang N, MacArthur DG, Wolde B, Onywera VO, Boit MK, et al. (2007) The ACTN3 R577X polymorphism in East and West African athletes. Med Sci Sports Exerc 39: 1985-1988.
5. Ahmetov II, Druzhevskaya AM, Astratenkova IV, Popov DV, Vinogradova OL, et al. (2008) The ACTN3 R577X polymorphism in Russian endurance athletes. Br J Sports Med 44: 649-652.
6. Druzhevskaya AM, Ahmetov II, Astratenkova IV, Rogozkin VA (2008) Association of the ACTN3 R577X polymorphism with power athlete status in Russians. Eur J Appl Physiol 103: 631-634.
7. Papadimitriou ID, Papadopoulos C, Kouvatsi A, Triantaphyllidis C (2008) The ACTN3 gene in elite Greek track and field athletes. Int J Sports Med 29: 352-355.
8. Roth SM, Walsh S, Liu D, Metter EJ, Ferrucci L, et al. (2008) The ACTN3 R577X nonsense allele is under-represented in elite-level strength athletes. Eur J Hum Genet 16: 391-394.
9. Massidda M, Vona G, Calo CM (2009) Association between the ACTN3 R577X polymorphism and artistic gymnastic performance in Italy. Genet Test Mol Biomarkers 13: 377-380.
10. Doring FE, Onur S, Geisen U, Boulay MR, Perusse L, et al. (2010) ACTN3 R577X and other polymorphisms are not associated with elite endurance athlete status in the Genathlete study. J Sports Sci 28: 1355-1359.
11. Muniesa CA, Gonzalez-Freire M, Santiago C, Lao JI, Buxens A, et al. (2010) World-class performance in lightweight rowing: is it genetically influenced? A comparison with cyclists, runners and non-athletes. Br J Sports Med 44: 898-901.
12. Ruiz JR, Arteta D, Buxens A, Artieda M, Gomez-Gallego F, et al. (2010) Can we identify a power-oriented polygenic profile? J Appl Physiol 108: 561-566.
13. Scott RA, Irving R, Irwin L, Morrison E, Charlton V, et al. (2010) ACTN3 and ACE genotypes in elite Jamaican and US sprinters. Med Sci Sports Exerc 42: 107-112.
14. Shang X, Huang C, Chang Q, Zhang L, Huang T (2010) Association between the ACTN3 R577X polymorphism and female endurance athletes in China. Int J Sports Med 31: 913-916.
15. Ahmetov II, Druzhevskaya AM, Lyubaeva EV, Popov DV, Vinogradova OL, et al. (2011) The dependence of preferred competitive racing distance on muscle fibre type composition and ACTN3 genotype in speed skaters. Exp Physiol 96: 1302-1310.
16. Chiu LL, Wu YF, Tang MT, Yu HC, Hsieh LL, et al. (2011) ACTN3 genotype and swimming performance in Taiwan. Int J Sports Med 32: 476-480.
17. Gineviciene V, Pranculis A, Jakaitiene A, Milasius K, Kucinskas V (2011) Genetic variation of the human ACE and ACTN3 genes and their association with functional muscle properties in Lithuanian elite athletes. Medicina (Kaunas) 47: 284-290.
18. Kothari ST, Chheda P, Chawla S, Chatterjee L, Chaudhry SK, et al. (2011) ACTN3 R577X polymorphism in Asian Indian athletes. International Journal of Human Genetics 11: 149-153.
19. Ruiz JR, Fernandez DVM, Verde Z, Diez-Vega I, Santiago C, et al. (2011) ACTN3 R577X polymorphism does not influence explosive leg muscle power in elite volleyball players. Scand J Med Sci Sports 21: e34-e41.
20. Sessa F, Chetta M, Petito A, Franzetti M, Bafunno V, et al. (2011) Gene polymorphisms and sport attitude in Italian athletes. Genet Test Mol Biomarkers 15: 285-290.
21. Eynon N, Ruiz JR, Femia P, Pushkarev VP, Cieszczyk P, et al. (2012) The ACTN3 R577X Polymorphism across Three Groups of Elite Male European Athletes. PLoS One 7: e43132.
22. Kikuchi N, Min SK, Ueda D, Igawa S, Nakazato K (2012) Higher Frequency of the Actn3 R Allele + Ace Dd Genotype in Japanese Elite Wrestlers. J Strength Cond Res.
23. Massidda M, Corrias L, Ibba G, Scorcu M, Vona G, et al. (2012) Genetic markers and explosive leg-muscle strength in elite Italian soccer players. J Sports Med Phys Fitness 52: 328-334.

**Part 2, excluded articles:**

*Necessary data cannot be obtained:*

1. Fatini C, Guazzelli R, Manetti P, Battaglini B, Gensini F, et al. (2000) RAS genes influence exercise-induced left ventricular hypertrophy: an elite athletes study. Med Sci Sports Exerc 32: 1868-1872.
2. Lucia A, Gomez-Gallego F, Chicharro JL, Hoyos J, Celaya K, et al. (2005) Is there an association between ACE and CKMM polymorphisms and cycling performance status during 3-week races? Int J Sports Med 26: 442-447.
3. Saunders CJ, September AV, Xenophontos SL, Cariolou MA, Anastassiades LC, et al. (2007) No association of the ACTN3 gene R577X polymorphism with endurance performance in Ironman Triathlons. Ann Hum Genet 71: 777-781.
4. Santiago C, Gonzalez-Freire M, Serratosa L, Morate FJ, Meyer T, et al. (2008) ACTN3 genotype in professional soccer players. Br J Sports Med 42: 71-73.
5. Gomez-Gallego F, Santiago C, Gonzalez-Freire M, Muniesa CA, Fernandez DVM, et al. (2009) Endurance performance: genes or gene combinations? Int J Sports Med 30: 66-72. (excluded for both ACE and ACTN3)
6. Juffer P, Furrer R, Gonzalez-Freire M, Santiago C, Verde Z, et al. (2009) Genotype distributions in top-level soccer players: a role for ACE? Int J Sports Med 30: 387-392.
7. Eynon N, Alves AJ, Yamin C, Sagiv M, Duarte JA, et al. (2009) Is there an ACE ID - ACTN3 R577X polymorphisms interaction that influences sprint performance? Int J Sports Med 30: 888-891.
8. Ahmetov II, Williams AG, Popov DV, Lyubaeva EV, Hakimullina AM, et al. (2009) The combined impact of metabolic gene polymorphisms on elite endurance athlete status and related phenotypes. Human Genetics 126: 751-761.
9. Santiago C, Ruiz JR, Muniesa CA, Gonzalez-Freire M, Gomez-Gallego F, et al. (2010) Does the polygenic profile determine the potential for becoming a world-class athlete? Insights from the sport of rowing. Scand J Med Sci Sports 20: e188-e194. (excluded for both ACE and ACTN3)
10. Ash GI, Scott RA, Deason M, Dawson TA, Wolde B, et al. (2011) No association between ACE gene variation and endurance athlete status in Ethiopians. Med Sci Sports Exerc 43: 590-597.

*Duplicate population:*

1. Lucia A, Gomez-Gallego F, Santiago C, Bandres F, Earnest C, et al. (2006) ACTN3 genotype in professional endurance cyclists. Int J Sports Med 27: 880-884.
2. Ruiz JR, Gomez-Gallego F, Santiago C, Gonzalez-Freire M, Verde Z, et al. (2009) Is there an optimum endurance polygenic profile? J Physiol 587: 1527-1534. (excluded for both ACE and ACTN3)
3. Santiago C, Ruiz JR, Muniesa CA, Gonzalez-Freire M, Gomez-Gallego F, et al. (2010) Does the polygenic profile determine the potential for becoming a world-class athlete? Insights from the sport of rowing. Scand J Med Sci Sports 20: e188-e194.
4. Eynon N, Alves AJ, Meckel Y, Yamin C, Ayalon M, et al. (2010) Is the interaction between HIF1A P582S and ACTN3 R577X determinant for power/sprint performance? Metabolism 59: 861-865.
5. Kikuchi N, Ueda D, Min SK, Nakazato K, Igawa S (2012) The ACTN3 XX Genotype is Under-represented in Japanese Elite Wrestlers. Int J Sports Physiol Perform.

*Comparison between athletes*

1. Bell W, Colley JP, Evans WD, Darlington SE, Cooper SM: ACTN3 genotypes of Rugby Union players: distribution, power output and body composition. *Ann Hum Biol* 2012; 39**:** 19-27.

*Incorrect* *data*

1. Kothari ST, Chheda P, Chatterjee L, Das BR (2012) Molecular analysis of genetic variation in angiotensin I-converting enzyme identifies no association with sporting ability: First report from Indian population. Indian J Hum Genet 18: 62-65.

*Departure from HWE in control group*

1. Turgut G, Turgut S, Genc O, Atalay A, Atalay EO (2004) The angiotensin converting enzyme I/D polymorphism in Turkish athletes and sedentary controls. Acta Medica (Hradec Kralove) 47: 133-136.
2. Oh SD (2007) The distribution of I/D polymorphism in the ACE gene among Korean male elite athletes. J Sports Med Phys Fitness 47: 250-254.
3. Eynon N, Duarte JA, Oliveira J, Sagiv M, Yamin C, et al. (2009) ACTN3 R577X polymorphism and Israeli top-level athletes. Int J Sports Med 30: 695-698.
4. Gineviciene V, Pranculis A, Jakaitiene A, Milasius K, Kucinskas V (2011) Genetic variation of the human ACE and ACTN3 genes and their association with functional muscle properties in Lithuanian elite athletes. Medicina (Kaunas) 47: 284-290. (excluded only in analysis of ACE)
