# Supplementary material for: The Association of Sport Performance with ACE and ACTN3 Genetic Polymorphisms: A Systematic Review and Meta-Analysis
Source: PLoS One. 2013 Jan 24;8(1):e54685. doi: 10.1371/journal.pone.0054685 (PMC3554644; doi:10.1371/journal.pone.0054685)
Supplement: Table S3 — Sport discipline: Definition of sport discipline in included articles (DOC) [file pone.0054685.s003.doc]

**Table S3 sport discipline**

**Part 1, articles focused on *ACE***

| **No.** | **Study Name** | **Disciplines** | **Definition in articles** |
| --- | --- | --- | --- |
| 1 | Gayagay G, 1998 | Endurance | rowing |
| 2 | Myerson S, 1999 | Endurance | >=5000m running |
|  |  | Power | <=200m running |
|  |  | Mix | 400-3000m running |
| 3 | Taylor R, 1999 | Endurance | hockey, cycling, skiing, track and field, swimming, rowing, gymnastics, other |
| 4 | Alvarez R, 2000 | Endurance | cycling, marathon, cross-country running |
| 5 | Rankinen T, 2000 | Endurance | cross-country skiing, biathlon, Nordic combined, long-distance running, middle distance running, road cycling |
| 6 | Nazarov I B,2001 | Endurance | long distance athletes more than 20min |
|  |  | Power | short distance athletes under 1min |
|  |  | Mix | middle distance athletes 1-20min |
| 7 | Woods D, 2001 | Power | swimming <= 400m |
| 8 | Scanavini D, 2002 | Endurance | road cycling, track and field, cross-country skiing |
|  |  | Power | flat-water kayak racing times in 30-210s |
| 9 | Collins M, 2004 | Endurance | Triathlon |
| 10 | Scott R A, 2005 | Endurance | running from 3000m-marathon |
| 11 | Hruskovicova H,2006 | N/A | marathon, half-marathon, inline skating |
| 12 | Aim O,2007 | Endurance | marathon |
|  |  | Power | sprinter, 100-200m |
| 13 | Eynon N, 2009 | Power | running in 100-200m |
| 14 | Costa A M,2009 | Power | short distance swimming in 50-200m |
|  |  | Mix | middle distance swimming in 400-1500m |
| 15 | Cieszczyk P,2009 | Endurance | rowing |
| 16 | Papadimitriou I D, 2009 | Endurance | long distance running in 3000m-marathon, middle distance running in 800-1500m, triathlon, walking |
|  |  | Power | running in 100-400m, jumping, throwing, decathlon |
| 17 | Tobina T, 2010 | Endurance | long distance running >5000m |
| 18 | Shenoy S, 2010 | N/A | triathlon |
| 19 | Kim C. H, 2010 | Power | discus, hammer, javelin; high, long and triple jump; pole vault; 100m, 200m running; weightlifting |
| **No.** | **Study Name** | **Disciplines** | **Definition in articles** |
| 20 | Ruiz J R, 2010 | Endurance | endurance running, road cycling |
|  |  | Power | jumping, sprinter |
| 21 | Muniesa C A, 2010 | Endurance | endurance runner and cyclist and rower |
| 22 | Sessa F, 2011 | Power | sprinter, short distance swimming, volleyball |
|  |  | intermittent | football, basketball, hockey |
| 23 | Scott R A, 2010 | Power | running <400m, jumping, throwing |
| 24 | Kikuchi N, 2012 | Power | wrestling |
| 25 | Massidda M, 2012 | N/A | soccer |

**Part 2, articles focused on *ACTN3***

| **No.** | **Study Name** | **Disciplines** | **Definition in articles** |
| --- | --- | --- | --- |
| 1 | Yang N, 2003 | Endurance | long distance cycling, rowing, swimming >=400m, track >=5000m, cross-country skiing |
|  |  | Power | track <=800m, swimming <=200m, judo, short distance track cycling, speed skating |
| 2 | Niemi A K, 2005 | Endurance | running 800- marathon, walking |
|  |  | Power | field events |
| 3 | Paparini A, 2007 | Endurance | rowing |
| 4 | Yang N, 2007 | Endurance | 3000m-marathon |
|  |  | Power | running<=400m, 110m hurdle, long and trple jump |
| 5 | Druzhevskaya A M, 2008 | Power | apline skiing, artistic gymnastics, bodybuilding, figurte skating, icee hockey, jumping, powerlifting, running 100-400m, ski jumping, soccer, speed skating, swimming 50-100m, throwing, volleyball, weightlifting |
| 6 | Roth S M, 2008 | Power | bodybuilder, powerlifter |
| 7 | Papadimitriou I D, 2008 | Endurance | long distance running 3000m-marathon, middle distance running in 800-1500m, triathlon, walking |
|  |  | Power | sprinter in 100-400m, jumping, throwing, dacathlon |
| 8 | Ahmetov I I, 2008 | Endurance | biathlon, cross-country skiing, race walking, road cycling, rowing, swimming 800-2500m, triathlon, swimming 1500m, cycling 40000m, running 10000m |
| 9 | Massidda M, 2009 | N/A | gymnastic |
| 10 | Shang X, 2010 | Endurance | rowing, track >=5000m, marathon, long distance cycling, swimming>=400m |
| 11 | Muniesa C A, 2010 | Endurance | endurance runner and cyclist and rower |
| 12 | Doring F E, 2010 | Endurance | cross-country skiing, bi- and triathlon, cycling, running, and rowing |
| 13 | Scott R A, 2010 | Power | running <400m, jumping, throwing |
| 14 | Ruiz J R, 2010 | Endurance | endurance running, road cycling |
|  |  | Power | jumping, sprinter |
| 15 | Chiu L L, 2011 | Power | swimming<=400m |
| 16 | Ahmetov I I, 2011 | N/A | speed skating 500-10000m |
| 17 | Sessa F, 2011 | Power | sprinter, short distance swimming, volleyball |
|  |  | intermittent | football, basketball, hockey |
| 18 | Gineviciene V, 2011 | Endurance | very long distance >30min, long distance 5-30min, middle distance 45s-5min |
|  |  | Power | sprint, other |
| 19 | Ruiz J R, 2011 | Power | volleyball |
| 20 | Kothari S T, 2011 | N/A | N/A |
| **No.** | **Study Name** | **Disciplines** | **Definition in articles** |
| 21 | Eynon N, 2012 | Endurance | running 5000m-marathon, road cycling, rowing, swimming 800–1500m, cross-country skiing 15–50km, triathlon, skating >=5000m , walkers, swimming >200 m , duathlete and water polo |
|  |  | Power | weightlifting, sprinting <=200 m, track and field jumping, volleyball, ice hockey, skating <=1000 m, boxing, wrestling, swimming <=200 m, weightlifting, figure skating, shot putting, heavy event and taekwondo . |
| 22 | Massidda M, 2012 | N/A | soccer |
| 23 | Kikuchi N, 2012 | Power | wrestling |
